# Intradural Conus Medullaris Lipoma With Neurological Deficit: A Rare Occurrence

**DOI:** 10.7759/cureus.14053

**Published:** 2021-03-23

**Authors:** Anuj Gupta, Kuldeep Bansal, Kalyan Kumar Varma Kalidindi, Aayush Bhargava, Aditya Verma

**Affiliations:** 1 Department of Orthopedics and Spine, Max Super Speciality Hospital, New Delhi, IND; 2 Department of Spine Services, Indian Spinal Injuries Center, New Delhi, IND; 3 Department of Orthopedics, University College of Medical Sciences, Guru Teg Bahadur Hospital, New Delhi, IND; 4 Department of Orthopedics, Indian Spinal Injuries Center, New Delhi, IND

**Keywords:** lipoma, spine, intradural, rare, surgery

## Abstract

Intradural lipoma without spinal dysraphism is a rare occurrence. Most of them are asymptomatic but can also present with neurological deficits. A 54-year-old male patient presented to us with progressive weakness in both lower limbs for six months. On physical examination and radiological workup, intradural lipoma was diagnosed. Due to progressive neurological deficit, the patient was treated surgically. On 2.5 years of follow-up, the patient showed complete neurological recovery. Intradural lipomas can also present with the neurological deficit at any age and should be managed surgically if the deficit is progressive in nature. Surgical management has a good outcome if done within two years of onset of symptoms.

## Introduction

Intradural lipoma in the lumbar spine is a rare entity. They are well-known for spinal dysraphism. Lipomas not associated with spinal dysraphism comprise only 1% of all cases [1,2]. More commonly seen in young and middle-aged people. Though non-dysraphism-associated lipomas are commonly asymptomatic, if they are, they are expected to cause a mass effect like any other tumor.

Since lipomas without dysraphism are very rare, hence there is a scarcity of literature describing those lipomas causing the neurological deficit. We present a case of intradural lipoma at the lumbar region causing neurological deficit and managed surgically.

## Case presentation

**Figure 1:**
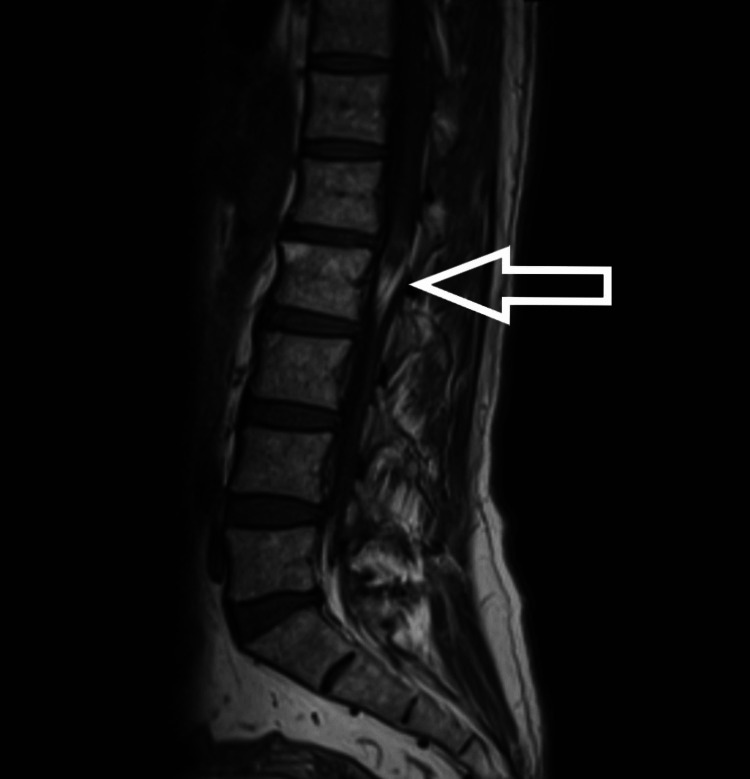
T1-weighted MRI (sagittal) showing hyperintense intramedullary mass

**Figure 2:**
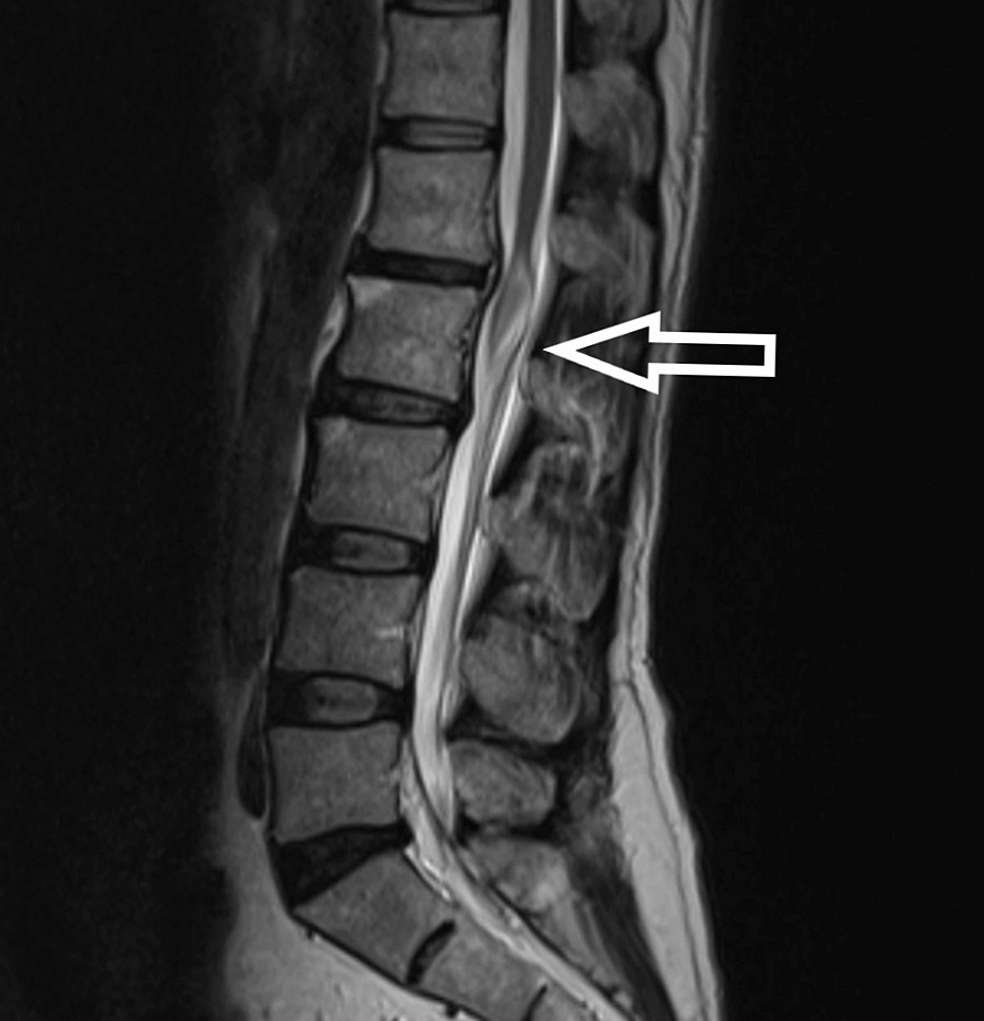
T2-weighted MRI (sagittal) showing hyperintense intramedullary mass

**Figure 3:**
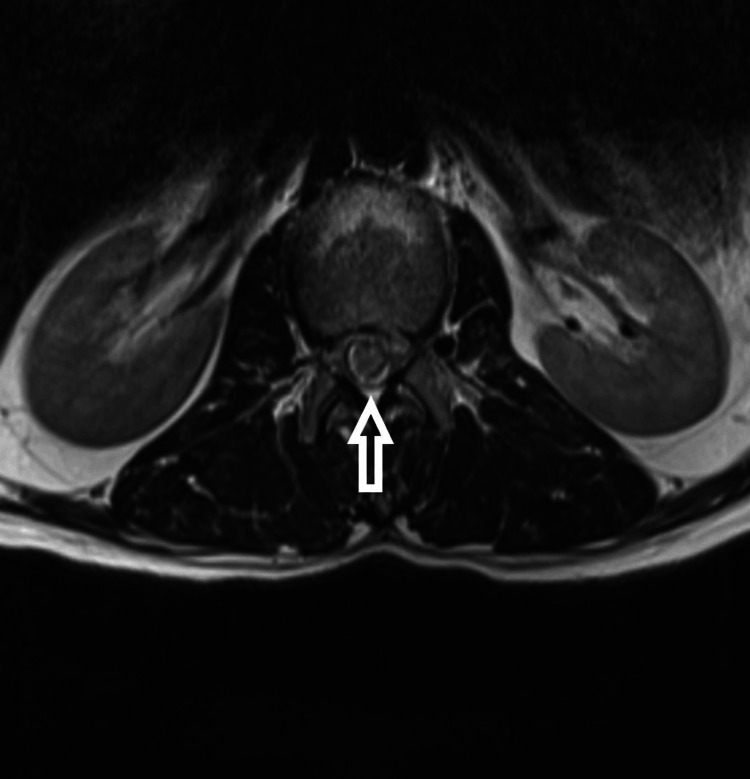
T1-weighted axial MRI

**Figure 4:**
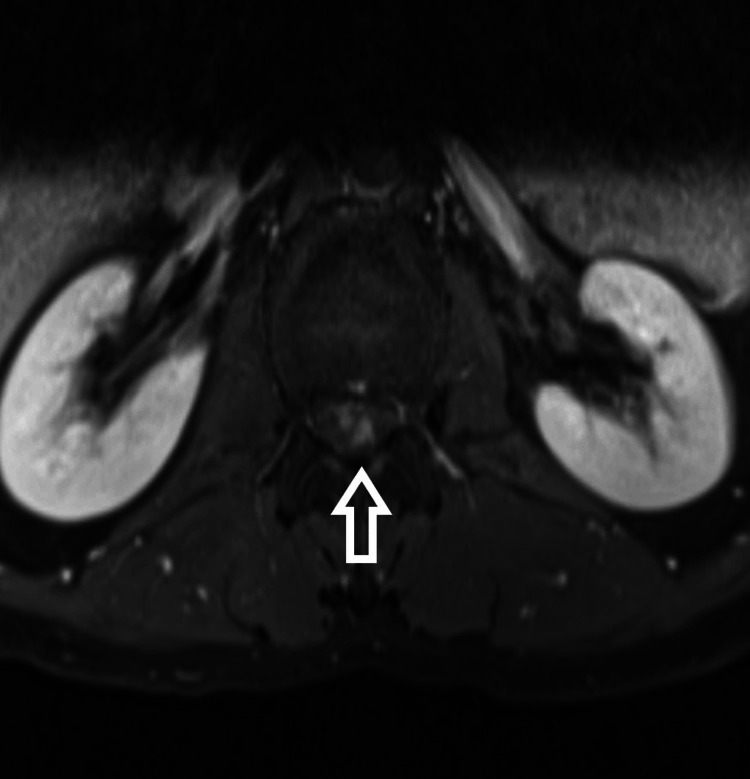
T2-weighted axial MRI

**Figure 5:**
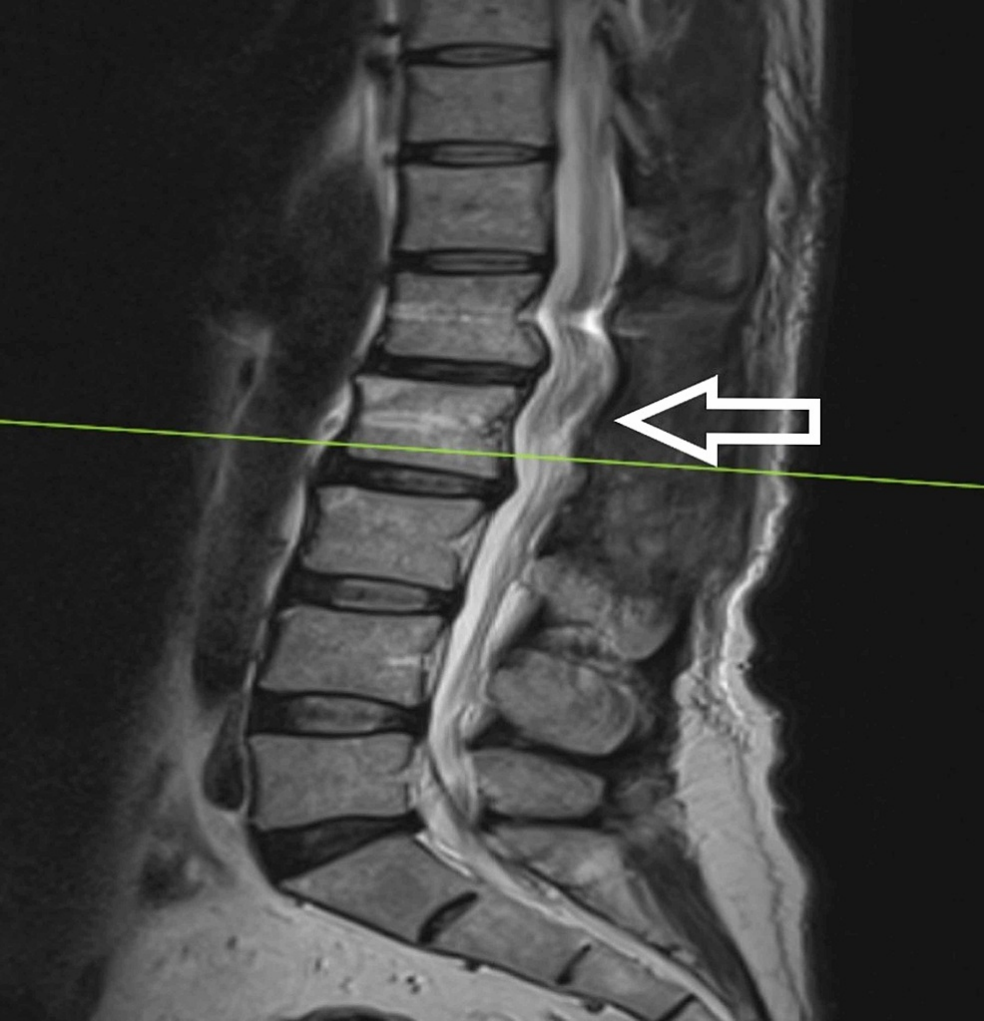
Immediate post-operative sagittal MRI

**Figure 6:**
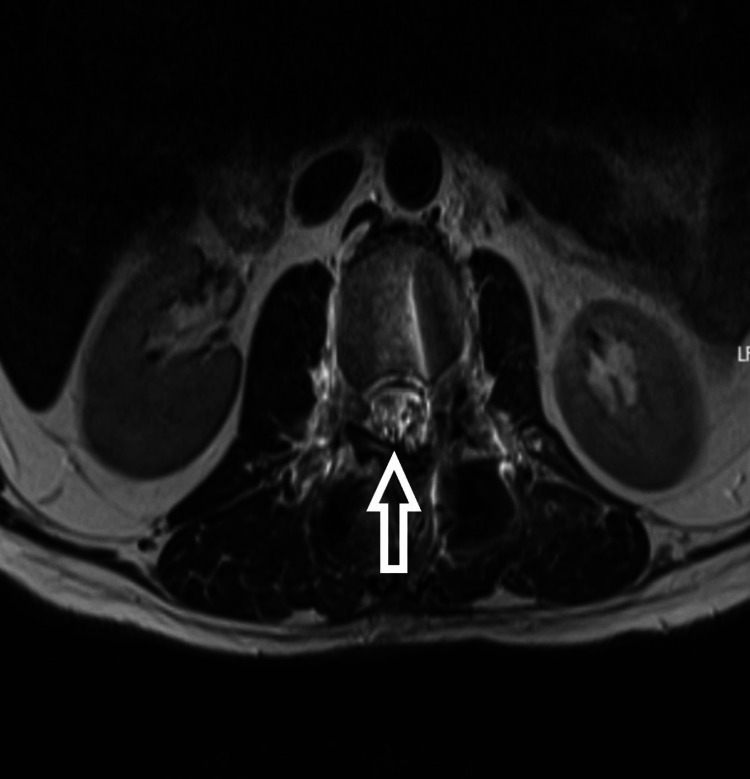
Immediate post-operative axial MRI

**Figure 7:**
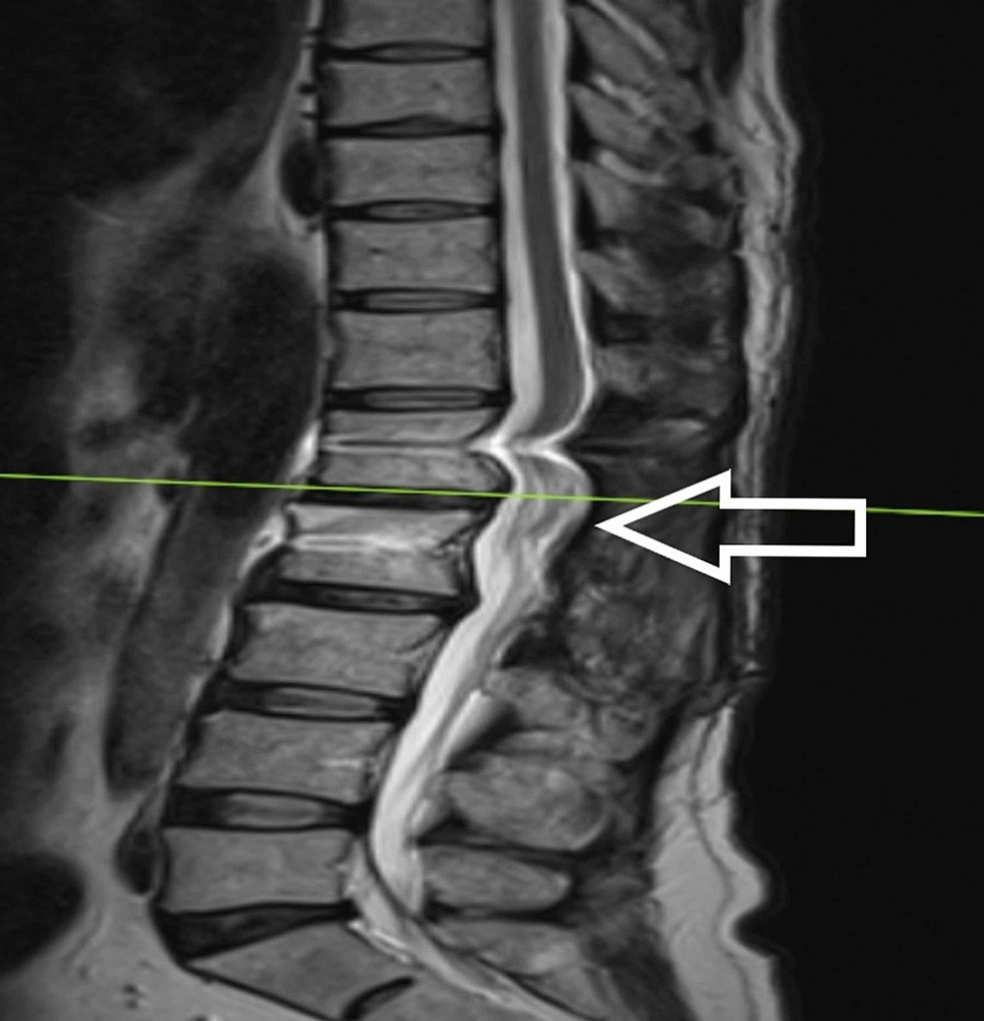
Sagittal MRI after 2.5 years

**Figure 8:**
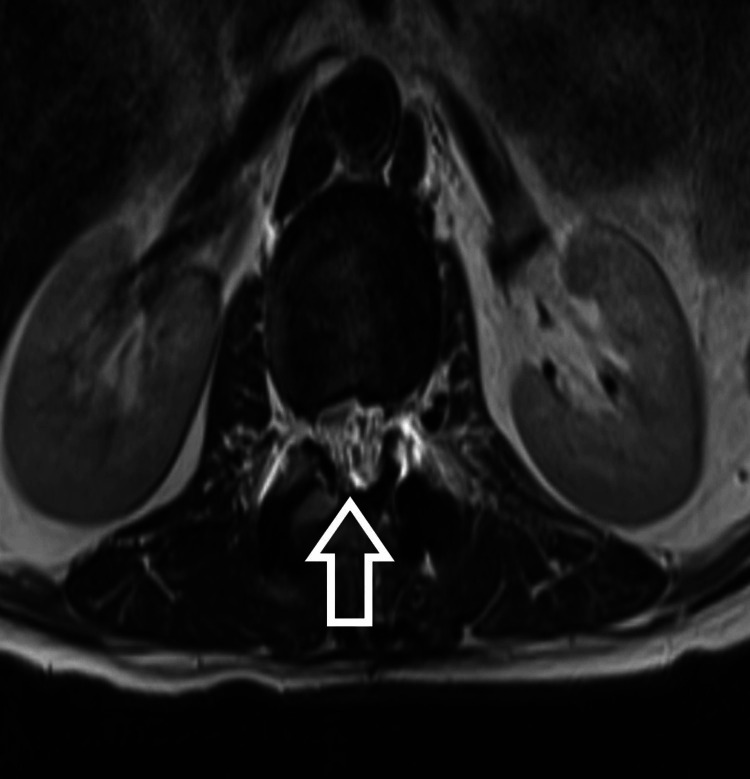
Axial MRI after 2.5 years

The patient is a 54-year-old male who presented to us with complaints of weakness in bilateral lower limbs for six months. The weakness was progressive in nature. There was no bladder and bowel involvement. On examination, there were three of five power in bilateral L3, L4, L5, and S1. The sensations were intact. The perianal examination was normal. MRI showed Intradural mass on dorsal aspect at L2-3 region or conus region and hyperintense on both T1 and T2- weighted images (Figures 1-4). A provisional diagnosis of lipoma was made on the basis of MRI. Considering the progressive nature of neurological deficit, surgical management was planned. A single-stage procedure was done wherein decompressive laminectomy with an exploration of lipoma and excision was done. On opening the dura, lipoma was placed in subpial space with tight adhesions with cauda equina nerve roots. Subtotal resection was done. Instrumentation was also done as stability was compromised due to wide laminectomy and removal of facets too. Post-operatively, the neurology of the patient was the same as pre-op. The patient was discharged on the fifth postoperative day and was kept on regular follow-up. A biopsy of the excised tissue was sent which confirmed the diagnosis of lipoma. On subsequent follow-up, the patient showed improvement in neurology (Figures 5, 6). At 2.5 years of final follow-up, the patient has complete recovery in neurological deficit (Figures 7, 8).

## Discussion

Lipoma in the lumbosacral spine without spinal dysraphism is a rare occurrence. The most common site of lipoma in the lumbosacral spine is the conus region [3]. Various theories have been postulated for the development of lipoma like metaplasia of adipose tissue in the pia membrane, a developmental error or proliferation of fat cells occasionally found in the pia membrane [4]. Though most of the lipomas are asymptomatic if actively growing, then they may mingle with nerve roots and meninges and may lead to neurological deficit [5]. This adhesion with nerve roots and meninges may lead to incomplete decompression of nerve roots during surgery or may even worse the neurological deficit. In our patient, the lipoma was located in the conus region, specifically at the L2-3 region.

MRI shows the presence of intradural mass at the L2-3 region which was intermingled with the nerve roots. The mass appeared hyperintense on both T1 and T2-weighted sequences. Such adhesions with the nerve root may be the cause of the progressive neurological symptoms of the patient and also it creates a difficult situation for the operating surgeon. Hence, proper surgical planning is essential as the safety margin of neurological preservation is thin [6].

As per the literature, the results are poor, in terms of neurological recovery, if the duration of symptoms is more than two years [7]. Our patient had symptoms of neurological involvement for the last six months and hence, he improved completely at 2.5 years of follow-up, which is in accordance with the literature.

Though the extent of resection does not correlate with the surgical result, attempts should always be made to resect as much as safely possible [8]. The laminectomy should be kept wide to safely access the mass and instrumentation should be used when stability is compromised. Since these masses encase the nerve roots, hence total resection is not possible. Studies have shown good results with partial removal of such mass [9,10].

## Conclusions

Intradural lipomas without spinal dysraphism are a rare occurrence and mostly asymptomatic. When presents with neurological involvement, surgical resection is necessitated. Wide laminectomy should be performed to have wide exposure and complete resection of mass should not be attempted as there are adhesions that can result in injury to nerve roots.
